# Fetal Growth Restriction: Does an Integrated Maternal Hemodynamic-Placental Model Fit Better?

**DOI:** 10.1007/s43032-020-00393-2

**Published:** 2020-11-19

**Authors:** F. Mecacci, L. Avagliano, F. Lisi, S. Clemenza, Caterina Serena, S. Vannuccini, M. P. Rambaldi, S. Simeone, S. Ottanelli, F. Petraglia

**Affiliations:** 1 Department of Biomedical, Experimental and Clinical Sciences, Division of Obstetrics and Gynecology, University of Florence, Viale Morgagni 44, 50134 Florence, Italy; 2 Department of Health Sciences, San Paolo Hospital Medical School, University of Milano, Milan, Italy; 3 Department of Molecular and Developmental Medicine, University of Siena, Siena, Italy

**Keywords:** Fetal growth restriction, Maternal hemodynamics, Cardiovascular diseases, Abnormal placentation, Cardiac output, Systemic vascular resistance

## Abstract

In recent years, a growing interest has arisen regarding the possible relationship between adverse pregnancy outcomes (APOs) and inadequate maternal hemodynamic adaptations to the pregnancy. A possible association between “placental syndromes,” such as preeclampsia (PE) and fetal growth restriction (FGR), and subsequent maternal cardiovascular diseases (CVD) later in life has been reported. The two subtypes of FGR show different pathogenetic and clinical features. Defective placentation, due to a poor trophoblastic invasion of the maternal spiral arteries, is believed to play a central role in the pathogenesis of early-onset PE and FGR. Since placental functioning is dependent on the maternal cardiovascular system, a pre-existent or subsequent cardiovascular impairment may play a key role in the pathogenesis of early-onset FGR. Late FGR does not seem to be determined by a primary abnormal placentation in the first trimester. The pathological pathway of late-onset FGR may be due to a primary maternal cardiovascular maladaptation: CV system shows a flat profile and remains similar to those of non-pregnant women. Since the second trimester, when the placenta is already developed and increases its functional request, a hypovolemic state could lead to placental hypoperfusion and to an altered maturation of the placental villous tree and therefore to an altered fetal growth. Thus, this review focalizes on the possible relationship between maternal cardiac function and placentation in the development of both early and late-onset FGR. A better understanding of maternal hemodynamics in pregnancies complicated by FGR could bring various benefits in clinical practice, improving screening and therapeutic tools.

## Introduction

Fetal growth restriction (FGR) takes place when the fetus does not achieve its biological growth potential [1]. Etiology of FGR can be various, including congenital malformations, and infectious or genetic anomalies, but in most cases, it is described as a consequence of impaired placental function [2]. This review focalizes on FGR where congenital malformations, and infectious or genetic anomalies were excluded. According to Delphi consensus, diagnosis of FGR is made on the basis of biometric and Doppler criteria, and it is subdivided in two subtypes: early- and late-onset FGR, according to the gestational age at presentation, considering 32 weeks as the established cut-off [3]. Beyond gestational age at presentation, the two subtypes of FGR also show different pathogenetic and clinical features. Early FGR is a rarer condition than late FGR and is more commonly associated with preeclampsia (PE) (60–70%) and abnormal placentation; it often ends up with poor fetal/neonatal outcomes including perinatal mortality. On the contrary, late FGR seems to be less related to PE and abnormal uterine circulation to the placenta [4].

## The Role of Placenta in FGR

The relationship between fetal growth and placental development has been extensively studied since the last century [5]. Throughout the first half of pregnancy, during the placentation process, the uterine maternal spiral arteries undergo extensive physiological modifications, related to the process of trophoblast proliferation, differentiation, and migration [6]: the extravillous trophoblast cells are crucial for spiral artery modification, determining the future placental development. Extravillous trophoblast develops during the early stage of pregnancy, when cytotrophoblast cells move away from the trophoblast columns of the anchoring villi and invade the maternal uterine tissues. The process of trophoblast differentiation and migration is related to the environmental oxygen content (systemic and/or local-intrauterine) [7]. During the invasion of the uterine wall, the endovascular trophoblasts, a subtype of extravillous trophoblast, invade the uterine spiral arteries and remodel their anatomy and function. Vascular changes occur with replacement of the arterial endothelium and media muscle cells of the vascular wall by endovascular trophoblasts. The replacement performed by the trophoblast leads to dilatation of the lumen of the vessels, loss of the muscular vascular component, and loss of vasomotor control. This process of arterial remodeling reduces maternal utero-placental blood-flow resistance and induces low-pressure and low-velocity utero-placental perfusion, according to the fetal demands [8]. Indeed, the velocity of maternal blood entering into the placental intervillous space determines the ability of perfusion of the villous tree, in order to obtain both an adequate development of the placental villi and an adequate transit time for the feto-maternal exchange. Moreover, the low pressure and velocity of flow obtained through the spiral artery remodeling prevent mechanical villous damage [9]. All these aspects allow an adequate placental development and functioning, leading in turn to an adequate feto-maternal exchange. Indeed, the physiology of the placentation process concurs with the physiology of the fetal growth.

However, signs of abnormal spiral artery remodeling may be observed in normal pregnancies, as well as normal spiral artery remodeling may be seen in cases affected by FGR [10].

Therefore, a non-placentocentric point of view should be considered to fully understand the pathophysiology of FGR and the two different phenotypes. The theory supporting the central role of the placenta in the genesis of FGR has the limitation of considering the placenta as a single item. However, its functioning depends on adequate systemic maternal perfusion; thus, maternal cardiovascular function is a relevant factor to acknowledge [11].

## Maternal Systemic Hemodynamic Adaptations in FGR

In recent years, there has been a growing interest on the possible relationship between FGR and inadequate maternal systemic hemodynamic adaptations to the pregnancy.

It is well known that maternal cardiovascular system physiologically changes during pregnancy. The major hemodynamic adaptations include increased cardiac output (CO), expanded blood volume, and reduced systemic vascular resistance (SVR) and blood pressure. SVR progressively drops from the first trimester and nadirs in the mid-second trimester ending in a plateau [12]. CO increases of about 20% at 8 weeks of gestations and continues to rise in a non-linear fashion reaching the peak in the early third trimester (+ 50% at 30–32 weeks) [13]. The increase in CO is determined by increased preload due to the rise in blood volume, increased maternal heart rate (HR) (10–30 bpm) and stroke volume (SV), and decreased afterload due to the reduced SVR [14].

**Fig. 1:**
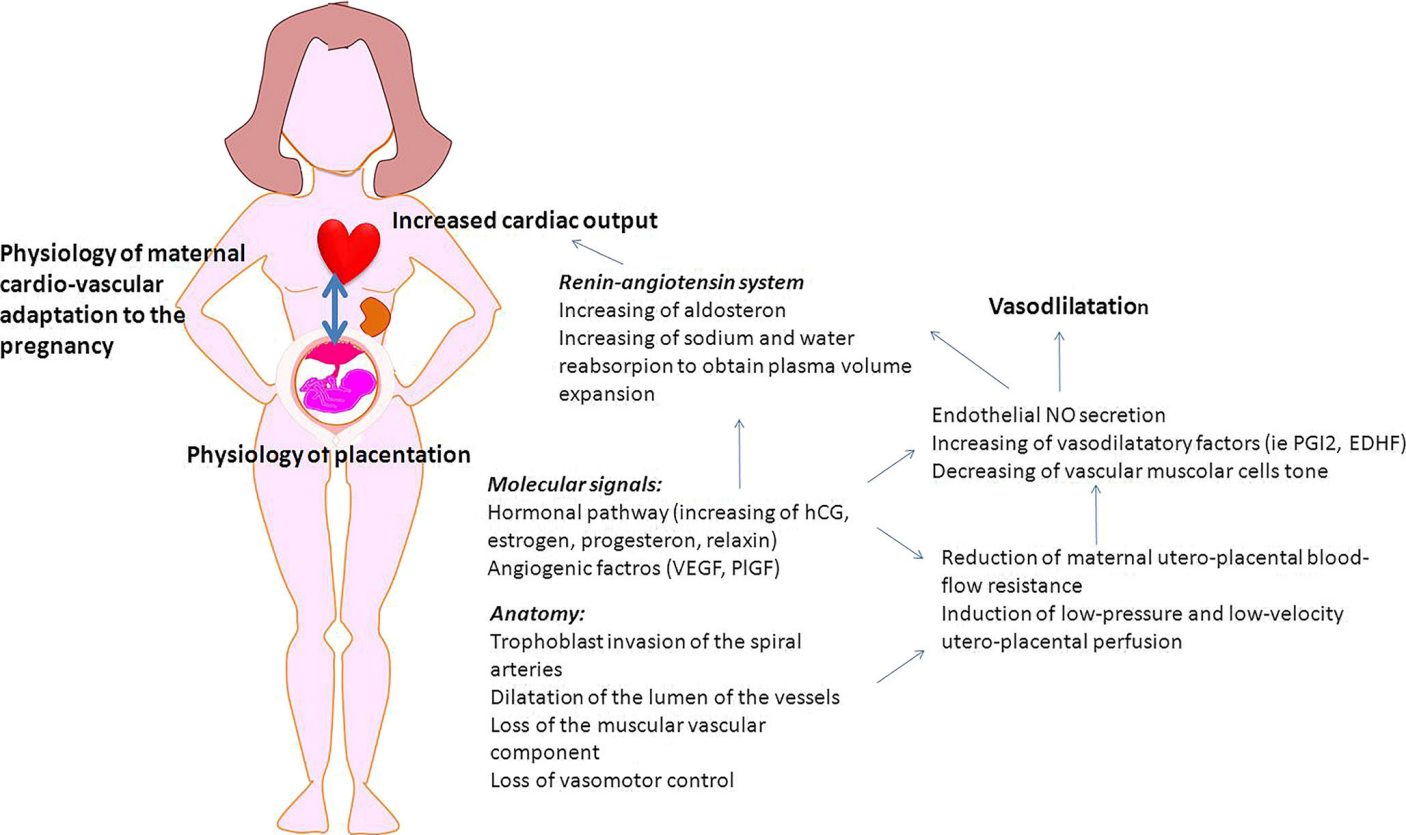
How placenta and maternal systemic cardiovascular systems interact for the women adaptation to the pregnancy. Spiral artery modification (obtained thanks to the trophoblast invasion process) reduces the utero-placental flow resistance. This artery modifications are associated with the placental secretion of angiogenic factors (i.e., vascular endothelial growth factor VEGF, placental growth factor PlGF) increasing nitric oxide (NO) and other vasodilatory factors such as prostacyclin (PGI2) and endothelium-derived hyperpolarizing factor (EDHF). Pregnancy hormones (human chorion gonadotropin HCG, estrogen, progesterone, relaxin) and placental secreted factors show many functions during pregnancy. They act as angiogenic factors and are able to activate the renin-angiotensin system, inducing the increase of aldosterone level and water and sodium reabsorption from the kidney, leading in turn to plasma volume expansion and hemodilution. Moreover, estrogens are indirectly able to intervene in the regulation of the pathway of NO and relaxin acts in the maternal hemodynamic adaptation, playing a role in the reduction of renal artery resistance. The increased NO in renal arteries also contributed to the reduction of renal vascular resistance. The result is an increased maternal blood volume with increased cardiac output and reduction in peripheral resistance. A detailed review of placental signaling of the maternal organism has been recently performed by Osol et al. [15]

During physiological pregnancy, a strict interaction occurs between placental development and maternal systemic adaptation to the pregnancy (Fig. 1) [15]: the decrease of SVR coincides with the reduction of utero-placental resistances; in fact, flow resistance in the uterine arteries decreases progressively during the first and second trimesters [16, 17].

Pregnancy is a challenge for women’s cardiovascular system, acting as a medical stress test for the mother [18]. Echocardiographic studies of uncomplicated pregnancies demonstrated an excessive increase in the left ventricular mass and remodeling. This is associated with diastolic dysfunction in a small but significant proportion of women at term, all of which revert to normal condition in the postpartum period [19, 20]. This is particularly evident in women who develop obstetric complications that, even if transient during the pregnancy state, could be seen as early indicators of a high-risk trajectory for future CVD [21].

Several studies have investigated the possible relationship between impaired hemodynamic adaptation during gestation, adverse pregnancy outcomes (APOs), and maternal cardiovascular diseases (CVD). A retrospective population study (CHAMPS study) [22] performed in Canada among 75,380 women with placental syndromes (gestational hypertension, preeclampsia, placental abruption, or placental infarction) demonstrated increased incidence of premature CVD in this population (HR 2.0, CI 1.7–2.2), with a higher risk in the combined presence of a maternal placental syndrome and FGR (HR 3.1, CI 2.2–4.5) or intrauterine fetal death (HR 4.4, CI 2.4–7.9) [22] .

In a recent review Lane-Cordova et al. summarized the role of APOs in the pathogenesis of later CVD [23]. They considered APOs as different syndromes that share similar pathogenesis of inadequate placentation, inflammation, and maternal vascular dysfunction. They also identified some risk factors and pathophysiology mechanisms which are shared by APOs and CVD. In this scenario, pre-existing maternal cardiovascular risk factors could increase the risk of placental vascular abnormalities leading to APOs. Those, in turn, could determinate persistent postpartum inflammation and cardiovascular dysfunction (abnormal cardiac mechanics, coronary endothelial dysfunction, loss of arterial compliance). All these risk factors, together with unhealthy behaviors such as sedentary lifestyle, smoking, and poor diet, cooperate to increase the risk of CVD following an APO.

Considering the different clinical phenotypes of early and late FGR and the heterogeneity of placental histology, this review focalizes on the possible role of maternal cardiac function in the genesis of both early and late-onset FGR.

## Early FGR

### Definition and Clinical Characteristics

According to Delphi consensus, the diagnostic criteria for early FGR are fetal abdominal circumference (AC) or estimated fetal weight (EFW) < 3nd centile or umbilical artery absent end diastolic flow (UA-AEDF) which are detected before 32 weeks of gestation. Alternatively, early FGR can be diagnosed if there are at least two of the subsequent three criteria: AC/EFW < 10th centile, uterine artery pulsatility index (UtA-PI) > 95th centile, and UA-PI > 95th centile [3].

The reported incidence of early FGR is about 0.5–1%. This condition is commonly associated with preeclampsia (PE) (60–70%) and abnormal placentation; it often results in poor fetal/neonatal outcomes, including perinatal mortality [4].

### Placenta in Early FGR

Defective placentation, due to a poor trophoblastic invasion of the maternal spiral arteries, is believed to play a central role in the pathogenesis of early-onset PE and FGR. The “placental theory” suggests that FGR may develop as a consequence of abnormal placentation with altered trophoblast invasion [2]. A failure of the physiological transformation of the spiral arteries, with persistence of a high-resistance and high velocity flow in the uterine circulation and intervillous space, is observed. Therefore, the consequences of defective trophoblast invasion may affect the final size of the placenta, the villous tree development, and the maternal to fetal transfer of oxygen and nutrients, resulting in FGR. In these conditions, the common placental findings are lesions consistent with maternal vascular malperfusion (MVM) [24]. MVM signs include a small placenta, histologic signs of decidual arteriopathy, placental infarcts, and the presence of histologic abnormalities of the placental villous tree, including distal villous hypoplasia. Un-remodeled or poorly remodeled spiral arteries may undergo to thrombosis of the vessels, potentially resulting in focal placental ischemia and infarct. Loss of functional placental parenchyma due to multifocal placental infarcts is associated with FGR with abnormal uterine artery Doppler waveforms [25]. Furthermore, the presence of hypoplastic, thin, and elongated villi [24, 26] is often associated with abnormal umbilical artery Doppler velocimetry [27]. Distal villous hypoplasia in mainly observed in pregnancy less than 32 weeks of gestation [24], consistently with the definition of early FGR.

The retention of the smooth muscular cells in the spiral artery walls may also cause intermittent perfusion leading to a stress response in the placenta. The stress related to placental malperfusion is likely to be an oxidative stress, secondary to the process of ischemia-reperfusion of intervillous space. It causes the release of a number of mediator factors, including pro-inflammatory cytokines, exosomes, and cell-free fetal DNA, into the maternal circulation, resulting in a maternal endothelial cells dysfunction, a systemic inflammatory response, and the clinical syndrome of PE [11, 28], including FGR. Moreover, the process of ischemia-reperfusion is associated to the development of syncytial knots in the placental villi, with alteration in the balance of angiogenetic agents: excess in the production and secretion of the antiangiogenic protein soluble fms-like tyrosine kinase (sFlt)-1 in syncytial knots [29] and suppression of syncytiotrophoblast secretion of the proangiogenic placenta growth factor (PlGF) [30, 31] are associated with early-onset FGR, with an abnormal maternal circulating level of angiogenic ratios of sFlt-1/PlGF; this abnormality correlates with the extent of placental MVM [32]. Abnormal placentation with abnormal angiogenesis and vasculogenesis is the rationale of the clinical trial protocol designed by the EVERREST Consortium. It aims to improve the outcome of severe early-onset fetal growth restriction administering maternal vascular endothelial growth factor (VEGF) gene therapy, via maternal uterine arteries [33]. VEGF is expressed in villous cyto- and syncitio-trophoblast and extravillous and endovascular trophoblast [34, 35]. It is normally secreted by the human placenta. Isoforms of secreted VEGF mediates the endothelial cell proliferation and endothelial tube formation, leading to branching angiogenesis [27]. The early-onset FGR is affected by a shift towards an antiangiogenic state with a reduction of maternal circulating VEGF, as well as the abovementioned reduction of PlGF and increasing of sFlt-1 [36, 37]. The promising aim of the EVERREST trial is to increase the local VEGF availability, to increase endothelial cell proliferation in the perivascular adventitia improving in turn the maternal spiral arteries vascular remodeling [38].

However, all these circulating placental mediators and the placental theory alone do not explain the correlation between APOs and maternal cardiovascular diseases. In particular, the placental theory does not explain why women who had PE in their pregnancies have a higher cardiovascular risk later in life or why women with pre-pregnancy cardiovascular risk factors have a higher risk of PE and FGR in pregnancy [39].

### Early FGR: a Model Based on Maternal Cardiovascular System Maladaptation and Placenta

There are two not mutually exclusive hypotheses associating placental abnormalities and maternal cardiovascular system adaptation in early FGR. The first one places the placenta as the primary cause of hemodynamic changes. High placental vascular resistance increases maternal uterine artery impedance, which contributes to an increase in maternal peripheral vascular resistance. This increase in maternal cardiac afterload opposes maternal cardiac output [40]. Therefore, the absence of an adequate maternal cardiovascular compensatory response to an abnormal trophoblastic invasion might determine a reduced placental perfusion and the development of FGR [41, 42].

The second hypothesis supports the role of a pre-pregnancy low maternal CO/high SVR causing reduced placental perfusion. This results in trophoblast impairment and triggers the consequences on the fetal and maternal side of the placenta. This sequence is compatible with the idea of maternal cardiovascular rather than primary placental dysfunction being the origin of complicated pregnancy [17]. This hypothesis is supported by the finding that a hypovolemic state, commonly observed in FGR pregnancies, also exists before the onset of the disease, both in early pregnancy [43–45] and in the preconception period. A recent study by Foo et al. longitudinally assessed cardiovascular function in 356 spontaneously conceived pregnancies in apparently healthy women, starting from preconception [46]. In this series, women with pregnancies complicated by FGR (both with and without PE) showed a reduced CO and elevated SVR prior to pregnancy, suggesting a hemodynamic dysfunction that exists before pregnancy and precedes any sequalae of placental disorder. Therefore, a suboptimal pre-pregnancy cardiovascular function could predispose women to impaired uterine artery blood flow and poor trophoblast development. However, since the number of patients was very limited (15 patients, including 3 cases of PE, 8 cases of FGR, and 4 cases of PE with FGR) and most FGR cases were late-onset forms, it is not possible to conclude that poor maternal cardiovascular performance is the cause of poor trophoblastic invasion [46]. It is possible that a maternal hemodynamics dysfunction may create a subclinical hypoxia. The maternal systemic hypoxic state may interfere with the process of cytotrophoblast differentiation towards extravillous trophoblast. This alteration could affect the development of endovascular trophoblast and, thereby, the spiral artery remodeling. In fact, during normal embryo and placenta development, there is an oxygen-dependent [7] phenotypic differentiation: first of all, in the morula, the differentiation towards the trophoblast lineage; second, the differentiation towards syncytio- and cyto-trophoblast; finally, the differentiation towards villous and extravillous trophoblast [47]. The cytotrophoblast differentiation towards extravillous trophoblast culminates in an invasion of maternal spiral arteries and vascular remodeling. Maternal oxygen deprivation may interfere with the trophoblast differentiation process, impairing in turn the utero-placental vessel development and spiral artery remodeling [7].

**Fig. 2:**
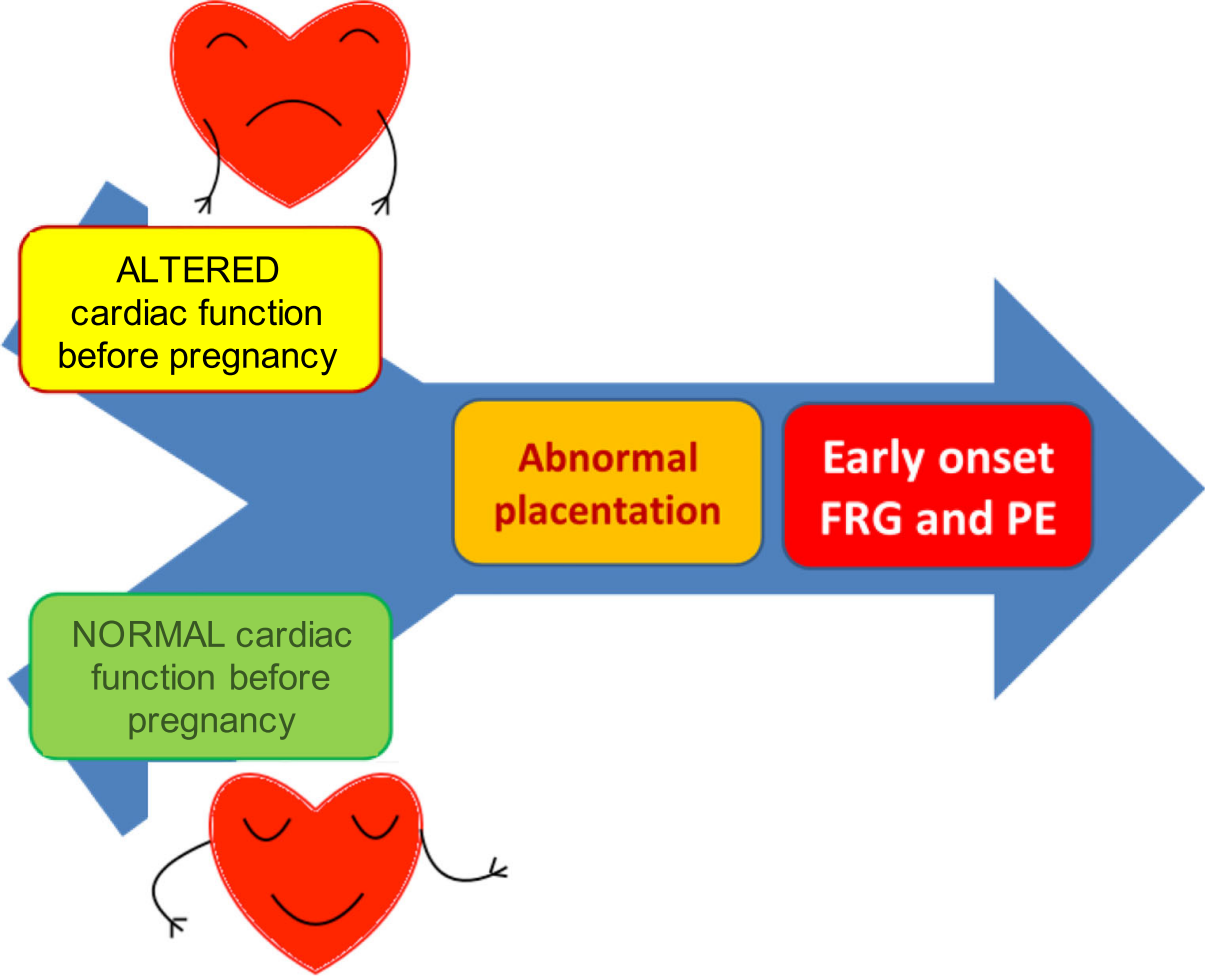
Pathogenetic hypotheses of early-onset FRG. In the pathogenesis of early FGR, pre-pregnancy cardiovascular dysfunction and abnormal placentation could coexist

Actually, the two hypotheses probably coexist (Fig. 2). The periconceptional cardiovascular status is characterized by a low cardiac reserve that, together with the impaired placental development, results in obstetric complications such as PE and early FGR. These pregnancies are characterized by a reduced expansion of maternal intravascular space, inadequate increase in preload and CO, and high SVR [40, 41, 48]. Although data on diastolic function are conflicting, pregnant women with early FGR seem to have higher prevalence of asymptomatic left ventricular diastolic dysfunction and impaired myocardial relaxation, compared to controls [41]. These cardiovascular findings may persist even in the post-partum period. In fact, there is growing evidence about asymptomatic left ventricular systolic and diastolic dysfunction, left ventricular hypertrophy, and prehypertension state in patients with previous early PE after delivery [49–51]. Notably, in agreement with this theory, Scholten et al. [52] calculated that the risk of recurrent PE and FGR in a subsequent pregnancy is inversely and linearly associated to pre-pregnancy plasma volume.

Moreover, it has been widely demonstrated that chronic hypertension is a risk factor for subsequent development of placenta-mediated complications such as FGR, superimposed PE, severe hypertension, and preterm birth (PTB) [53, 54]. Different types of chronic hypertension exist. The more frequent type is the “essential” hypertension (without a known cause), the other type (affecting less than 15% of patients) is the “secondary” hypertension (that is related to underlying condition such as renal, endocrine, and vascular disease). Both these types could affect the pregnancy [55]. The incidence of superimposed pregnancy hypertension disorder on preexisting chronic hypertension increases with the severity of preexisting hypertension (severe versus mild) and with a poor pharmacological control [56]. Chronic hypertension, and PE, is considered an indication to low-dose aspirin use for prophylaxis of PE itself and FGR [57, 58]. Interestingly, the incidence of adverse fetal outcomes seems to be related with the duration and the severity of the preexisting chronic hypertension, reflecting the association with end-organ damage such as maternal cardiac dysfunction [59]. In women affected by severe chronic hypertension, end-organ disease, or secondary hypertension, the risk of abnormal fetal growth increases to 25–40% [60].

Other recent findings have led to the re-evaluation of the cause-effect inference between trophoblast development and spiral artery transformation, focusing on the role of maternal systemic hemodynamic rather than local placental development. It is well known that uterine artery blood flow Doppler assessment is a useful tool for the prediction of early-onset PE and FGR. The relationship between uterine artery blood flow and trophoblast cell function is considered to be a proxy of trophoblast invasion, indicating that the persistence of high resistance in the uterine artery Doppler indices reflects an impaired trophoblast invasion and inadequate spiral artery remodeling. A modern point of view suggests an inverse biological association: maternal poor hemodynamic adaptation to pregnancy leads to increased uterine resistance and, in turn, poor placental perfusion may result in impaired trophoblast invasion and function [61].

## Late FGR

### Definition and Clinical Characteristics

Late FGR is diagnosed after 32 weeks of gestation by an AC/EFW below the 3rd centile or by at least two of the following three criteria: AC/EFW < 10th centile, AC/EFW centile crossing more than two quartiles on growth curves, cerebroplacental ratio (CPR) < 5th centile, or UA-PI >95th centile [3].

Late FGR is rarely associated with other placental disease such as PE (15%) and is related with a low perinatal mortality rate. However, this is a more frequent condition with an estimated prevalence of 5–10% and its detection and diagnosis are still a challenge for physicians. Therefore, late FGR has a significant clinical impact in terms of fetal outcomes [4].

### Placenta in Late FGR

Unlike the early phenotype, late FGR does not seem to be primarily determined by abnormal placentation in the first trimester [4]. However, histopathological examinations of placentas from FGR fetuses delivered at term demonstrate a higher rate of vascular lesions (especially infarcts and thrombotic event) compared to normal-term pregnancies. These histological findings are similar to those of preterm FGR, suggesting a quantitative difference in severity and extension of lesions rather than qualitative. According to Kim et al. [62], failure of the physiological transformation of the maternal spiral arteries is not an “all or nothing” event in every vessel; therefore, different amount of abnormal remodeling might imply different severity and different time of the onset of the disease. Moreover, previous studies suggested a gradient of effect of trophoblast invasion along the spiral arteries, with absence of remodeling in the decidual or myometrial segment, or both. More severe FGR occurs in cases of abnormal deep invasion as opposed to decidual [63].

Supporting this hypothesis, neither early assessment of placental function nor second-trimester uterine artery Doppler velocimetry screening in the general population has the ability to predict late-onset FGR. Uterine artery Doppler impedance classically is thought to represent placental development through spiral artery invasion: high impedance reflects inadequate trophoblast invasion and narrow spiral arteries. It is a useful screening for early-onset PE and FGR, but not for late-onset phenotypes.

Accordingly, in 2016, a multicenter randomized trial among 11,667 pregnant women demonstrated that routine second-trimester uterine artery Doppler ultrasound in non-selected population has low sensitivity in detection of late FGR (24%) and SGA fetuses (18%) [64].

Moreover, it has been suggested that the hypoxic status observed in the placenta of late FGR is not related to defective trophoblast invasion but it is mainly determined by subsequent intervillous malperfusion and reduced intraplacental oxygen concentration [65–67]. Increased abnormal development of the placental villous tree may be observed in these pregnancies, with late FGR and normal umbilical artery Doppler waveforms, in association with accelerated villous maturation [27]. In those cases, placental villi are small, short, and hypermature for gestational period, associated with an increase in syncytial knots [24]. These characteristics contribute to reduce vascular impedance and explain why in most cases umbilical artery Doppler waveforms are normal in these cases of FGR [27].

The placenta of late-onset FGR may also show completely different late-onset histological findings with delayed villous maturation. This is a placental histopathologic finding that can be found near the end of pregnancy and rarely before 34 weeks of gestation [24]. It is clinically associated with FGR as well as intrauterine hypoxia, maternal metabolic disorder, and obesity [68]. It is characterized by a monotonous unvaried villous population, with increased villous diameter, with centrally placed capillaries, cellular stroma, a continuous cytotrophoblast layer, and reduced numbers of vasculosyncytial membranes [24].

All considered, it is challenging to identify methods for the early prediction of late-onset FGR and there is no strong evidence of the best approach for its detection and management [58].

### Late FGR: a Model Based on Maternal Cardiovascular System Maladaptation and Placenta

As previously stated, Doppler velocimetry assessment of uterine arteries during the first trimester of pregnancy is not a sensitive method for predicting the risk of late-onset FGR. Recently, Binder et al. [69] evaluated 5887 pregnancies with longitudinal uterine Doppler assessment along the third trimester. It was demonstrated that one-third of patients show a de novo increase in uterine artery resistance in the late third trimester, having previously exhibited normal indices. Moreover, this group had a higher prevalence of SGA baby and lower birthweight centile. These variations may suggest that the maternal systemic and uterine vascular resistance in pregnancy changes independently of the direct consequences of placental invasion. Therefore, evaluating maternal hemodynamic changes in pregnancy seems to be a key factor in recognizing women at higher risk to have late FGR.

It has been suggested that pregnancy may unmask a preexisting sub-clinical maternal cardiac dysfunction. Thus, a subclinical impairment of maternal cardiac function existing before pregnancy may emerge during the strain of pregnancy, failing the adaptation of maternal cardiovascular system to the feto-placental demands [70, 71]. Therefore, the pathological pathway of late-onset FGR may not be wholly due to the failure of angiogenesis and physiological low-resistance trophoblast invasion, rather to a primary cardiovascular lack of adaptation with low CO and SV and high SVR. In late-FGR, these parameters do not show physiological changes, showing a flat profile through the trimesters of pregnancy, remaining similar to those of non-pregnant women [72].

In a prospective observational cohort study, Stott et al. [72] assessed periodically maternal hemodynamic parameters among 84 high-risk pregnant women. In pregnancies with neonatal birthweight ≥ 10th percentile, they found a longitudinal maternal hemodynamic variable pattern consistent with the known physiological changes in pregnancy. On the contrary, in pregnancies affected by late-onset FGR, the authors reported a static pattern with lower CO and SV and higher SVR. This primary and persistent failure of the maternal cardiovascular system to adapt to pregnancy may lead to abnormal placental villous tree development, i.e., delayed villous maturation, as occurs in late FGR.

Therefore, assessment of maternal hemodynamic rather than uterine artery Doppler in the second trimester could improve to identify those women at higher risk to develop late FGR. Using multivariate logistic regression analysis, Roberts L.A. et al. demonstrated that prediction of birthweight < 3rd percentile is improved by assessment of maternal hemodynamic function along with maternal history and demographics, fetal biometry, and Dopplers [73]. Especially in high-risk pregnancies, such as those complicated by previous PE, chronic hypertension, or diabetes, the combination of maternal anthropometric and hemodynamic parameters assessed early in the first trimester of pregnancy can provide a screening tool for late FGR [74].

The maternal hemodynamic variables, longitudinally assessed in late FGR and adequate for gestational age (AGA) fetuses, differed from the first to the third trimester of gestation. Moreover, the highest difference in cardiovascular parameters was at 25 weeks [75], suggesting that a screening at this gestation may have an even more robust predictive potential.

**Fig. 3:**
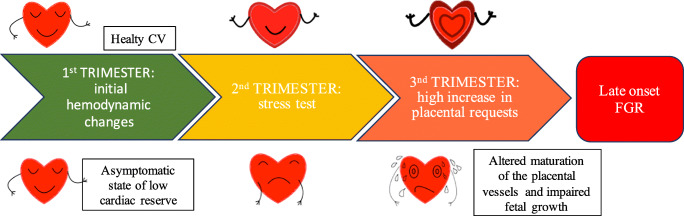
Pathogenetic hypotheses of late-onset FRG. How placenta responds to the pump deficit? At about 25 weeks of gestation, there are maximal divergences between the performance of a healthy CV system and one with a low cardiac reserve

Based on these evidences, the maternal cardiovascular system in late-onset FGR becomes unable from the second trimester onwards to respond to the increasing demands of pregnancy, when the placenta, normally developed until that moment, increases its requests. The hypoperfusion and maladaptive response of the placenta could be the consequence of a primary pump deficit, which leads to an altered maturation of the placental villous tree and therefore to an altered fetal growth [72, 75] (Fig. 3).

## Maternal Cardiac Function Assessment to Distinguish FGR from SGA

Maternal hemodynamic indices are relevant both for screening and diagnosis of FGR, helping to distinguish between a small for gestational age (SGA) and a growth-restricted fetus [40, 45].

In a recent prospective study [76], a single hemodynamic investigation using a non-invasive device (USCOM-1A®) was made at the time of diagnosis of FGR or SGA in normotensive pregnancies. Pregnancies complicated by FGR presented with worse maternal hemodynamic function, as evidenced by lower HR and CO as well as higher mean artery blood pressure, SVR, and uterine artery resistance, when compared to pregnancies with SGA fetus or healthy control pregnancies. SV was similar in the pregnancies complicated by FGR to those with a SGA fetus or healthy control pregnancies. Since CO is determined by multiplying SV and HR, the authors suggested that the observed difference in maternal CO was a consequence of lower maternal HR. Normally, relative tachycardia may be a physiological response to the increasing metabolic demands of pregnancy. An increased HR helps to maintain CO against SVR, thus ensuring adequate placental blood flow. When this adaptation fails to occur, FGR develops. In SGA pregnancies without FGR, there were no differences in maternal hemodynamic indices compared to healthy control pregnancies, supporting the hypothesis that these are normally grown babies who have met their growth potential.

Consistent results are derived from a retrospective study among 51 pregnancies with FGR or SGA fetuses [77]. Women with FGR had lower CO and higher SVR compared to women with SGA fetuses and even to controls. On the contrary, there was no difference in maternal hemodynamics between SGA and control group. Using multivariate analysis, the authors found that CO at diagnosis was the main independent predictive factor for the length of stay in neonatal intensive care unit for newborns.

## Maternal Cardiovascular System and Placenta: the Vicious Cycle

As the pre-pregnancy cardiac function is related to the placenta function, so does the placenta with the post-pregnancy cardiovascular state. It has been suggested that defective placentation and the resultant proinflammatory state are characterized by an antiangiogenic state and endothelial and cardiac maternal dysfunction; this persists in the postpartum and may underlie development of CVD later in life. In fact, maternal cardiac dysfunction was also found in non-pregnant women with a history of PE and/or FGR. Moreover, in these women, a new pregnancy might worsen an impaired hemodynamic environment, which can predispose to the recurrence of similar complications [23].

In 2016, Valensise et al. found signs of systolic and diastolic dysfunction in the preconception state before a second pregnancy complicated by recurrent early PE. Moreover, previous early preeclamptic patients with non-recurrent PE showed left ventricular structural and functional features intermediate compared to controls and recurrent PE [51].

A large Norwegian register–based study [78] reported an association between PE and the subsequent risk of major coronary events (OR 2.1, CI 1.73–2.65). The risk was markedly increased when PE was combined with SGA fetuses (OR 3.3, CI 2.37–4.57) or PTB (OR 5.4, CI 3.74–7.74). The highest risk for coronary events was found for women with recurrent PE complicating the first 2 pregnancies.

Gestational age at delivery and fetal growth seem to correlate with subsequent risk of maternal cardiovascular disease regardless of PE, with a risk of coronary heart disease that is markedly increased in women with most serious and early FGR cases [79].

In addition, endothelial dysfunction in the postpartum state, demonstrated by impaired endothelium-dependent vasodilatation and increased arterial stiffness, was found in women with previous early-onset PE and FGR, but not in women with previous late-onset PE [80, 81]. A history of HELLP syndrome, FGR, and early-onset PE seems to classify a subgroup of women with higher risk for future endothelial dysfunction and CVD [82].

From a clinical point of view, some FGR-linked histological lesions of decidual arteriopathy share similar aspects with CVD, representative of systemic and local inflammatory milieu. During pregnancy, a fine maternal immune system regulation is necessary to control fetal growth [83] and to allow the implantation and placentation process. The extravillous trophoblast cells encounter and interact with cells in the decidual stroma, including macrophages, T cells, and uterine natural killer cells. The extravillous trophoblast and natural killer cells cooperate to release proteases and cytokines that stimulate the spiral artery modifications [84, 85]. Local decidual maternal-fetal cell immune dysregulation predisposes to abnormal spiral artery remodeling and acute atherosis, with fibrinoid necrosis and accumulation of a subtype of macrophages, forming the foam cells, with or without perivascular inflammatory infiltrate. It has been suggested that acute atherosis is an inflammatory lesion, similar to the systemic and cardiac atherosclerosis out of pregnancy [86].

This represents a vicious circle: pre-pregnancy cardiovascular risk factors, together with innate immune dysregulation and genetic predisposition, may contribute to poor placentation. Incomplete placentation results in placental ischemia and release of antiangiogenic and proinflammatory factors causing cardiovascular damage during pregnancy and in post-partum period [23].

## Future Frontiers of Maternal Hemodynamics: Emerging Treatments of FGR

There are no proven treatments for FGR able to improve fetal growth in utero. The only effective therapeutic option is timely iatrogenic PTB after administration of maternal corticosteroids and magnesium sulphate to improve neonatal outcome [87].

Several studies showed that the prophylactic use of low-dose aspirin in women at high risk of developing PE and FGR may reduce the prevalence of these complications [88, 89]. Aspirin has a number of vascular and coagulation effects supporting FGR prevention: it suppresses the production of prostaglandins and thromboxane, inhibiting platelet aggregation; promotes nitric oxide release from the vascular endothelium [90]; increases the activity of heme oxygenase-1 in endothelial cells to catabolize heme, which leads to a reduction in oxidative stress, injury, and inflammation [91]. Most national and international guidelines recommend a 100–150-mg aspirin dose daily administered before16 weeks to prevent FGR in women at high risk [58].

The understanding of the possible role of maternal hemodynamics in the pathogenesis of FGR may suggest the development of new therapeutic tools based on this mechanism.

NO donors have been widely studied for both prevention [92] and management of PE and FGR and they seem to improve uterine and umbilical blood flow [93, 94]. NO is an autocrine and paracrine signaling molecule that is synthesized from l-arginine by a family of calcium-calmodulin-dependent enzymes called nitric oxide synthases (NOS). It is released by endothelial cells with a physiological vasodilating effect that increases blood flow. Moreover, it induces a decreased responsiveness to vasopressors and inhibition of platelet function [95]. As previously mentioned, in normal pregnancy, the trophoblast produces NO, which appears to play an important role in normal placenta development inducing a reduction in SVR of the fetoplacental and uterine circulations. On the contrary, in pregnancies complicated by PE or FGR, placental hypoxia and endothelial dysfunction are associated with decreased release of NO [44]. NO donors act on maternal hemodynamics with different mechanisms, including the reduction in blood pressure values, the increase in heart rate, and the dilatation of the capacitance vessels. The latter effect on the venous bed causes the increase of venous pooling and the decrease of the preload, which is already defective in mothers of FGR fetuses. In order to avoid this potential harmful effect, simultaneous plasma volume expander (PVE) administration seems to be a reasonable approach. In fact, fluid management induces an increase in preload, whereas NO donors act by reducing SVR and decreasing afterload, thus improving SV and CO. Valensise et al. [96] found that the combined therapy of PVE and NO donors in hypertensive mothers of severely growth-restricted fetuses improves maternal hemodynamic parameters (causing decrease in SVR and increase in CO) and reduces fetal-placental impedance (reappearance of end-diastolic flow in the UA), inducing prolongation of gestation. In a subsequent cohort study on FGR pregnancies, Tiralongo et al. [97] confirmed these results, reporting a significant increase in CO, SV, and a decrease of SVR after therapy with NO donors and PVE. In addition, an improvement of fetal growth was showed in the treated group. On the contrary, no differences were found in the untreated group.

Promising results have been recently obtained in case of statin administration. Statins are generally used for the inhibition of cholesterol biosynthesis and for the prevention of atherosclerotic cardiovascular diseases. Treatment with pravastatin 40 mg/die in pregnancies affected by early-onset FGR was associated with a significantly better angiogenic profile, improving the sFlit1/PlGf ratio [98]. This result could be related to the association between pleiotropic effect of statins and the NO signaling, as observed in animal models [99]. In human pregnancies, although the Doppler progression did not change with the pravastatin therapy, the time from diagnosis to delivery increased, with higher newborn birthweights [98].

Recently, sildenafil, a phosphodiesterase type 5 inhibitor, has been proposed for the treatment of FGR [100, 101]. Sildenafil enhances the actions mediated by NO, potentially improving placental perfusion. In a case-control study by von Dadelszen et al. [102], the use of sildenafil citrate in pregnancies complicated by severe FGR was associated with a significant increase in AC. However, the authors did not evaluate hemodynamic changes of maternal and fetal circulation. Subsequent studies have reported a decrease in UtA- and UA-PI in FGR fetuses with the use of sildenafil [103–105]. Khalil et al. [106] found that this molecule increased maternal HR and reduced blood pressure and arterial stiffness in pregnancies complicated by severe early-onset FGR. However, these CV effects were mild and short lasting. Moreover, sildenafil did not prolong the pregnancy duration and did not improve pregnancy outcomes in severe early-onset FGR, when tested in an adequately powered multicenter randomized controlled trial [100]. Furthermore, the Dutch STRIDER trial was prematurely stopped due to excess neonatal mortality secondary to pulmonary hypertension, possibly related to the therapy [101].

The identification of maternal hemodynamic status should be helpful in choosing the most appropriate treatment in women with hypertension and FGR, according to the underlying cardiovascular phenotype. In those cases, drugs with beta-blocking activity are negatively chronotropic and reduce CO. Therefore, they may be harmful in women with FGR in which the uteroplacental circulation is already impaired. Conversely, calcium antagonists are likely to be more effective in the high-SVR state that characterizes hypertensive women with FGR [107].

## Conclusion

Pregnancy represents a challenge to women’s cardiovascular system, acting as a medical stress test for the mother. [18] This pregnancy “stress” could unmask a state of limited reserves and subclinical impairment of maternal cardiac function at the time of conception. If the cardiovascular system fails to improve its function during pregnancy, it could be damaged, compromising fetal outcomes [70, 71]. In fact, FGR, with or without coexisting PE, is associated with low CO and high SVR maternal phenotype, irrespective of gestation.

As far as recent evidence suggests, both early and late FGRs are associated with placental disease, albeit with different pathways. In the early-onset FGR, the placental abnormal development seems to be the cause of FGR and is associated with histological signs of abnormal early implantation (abnormal trophoblast invasion and inadequate spiral artery remodeling). In the late-onset FGR, it is unclear whether placental abnormalities are related to less severe forms of abnormal placentation at early pregnancy or to late placental damage occurring during the second half of pregnancy (such as abnormal development and maturation of placental villous tree).

**Fig. 4:**
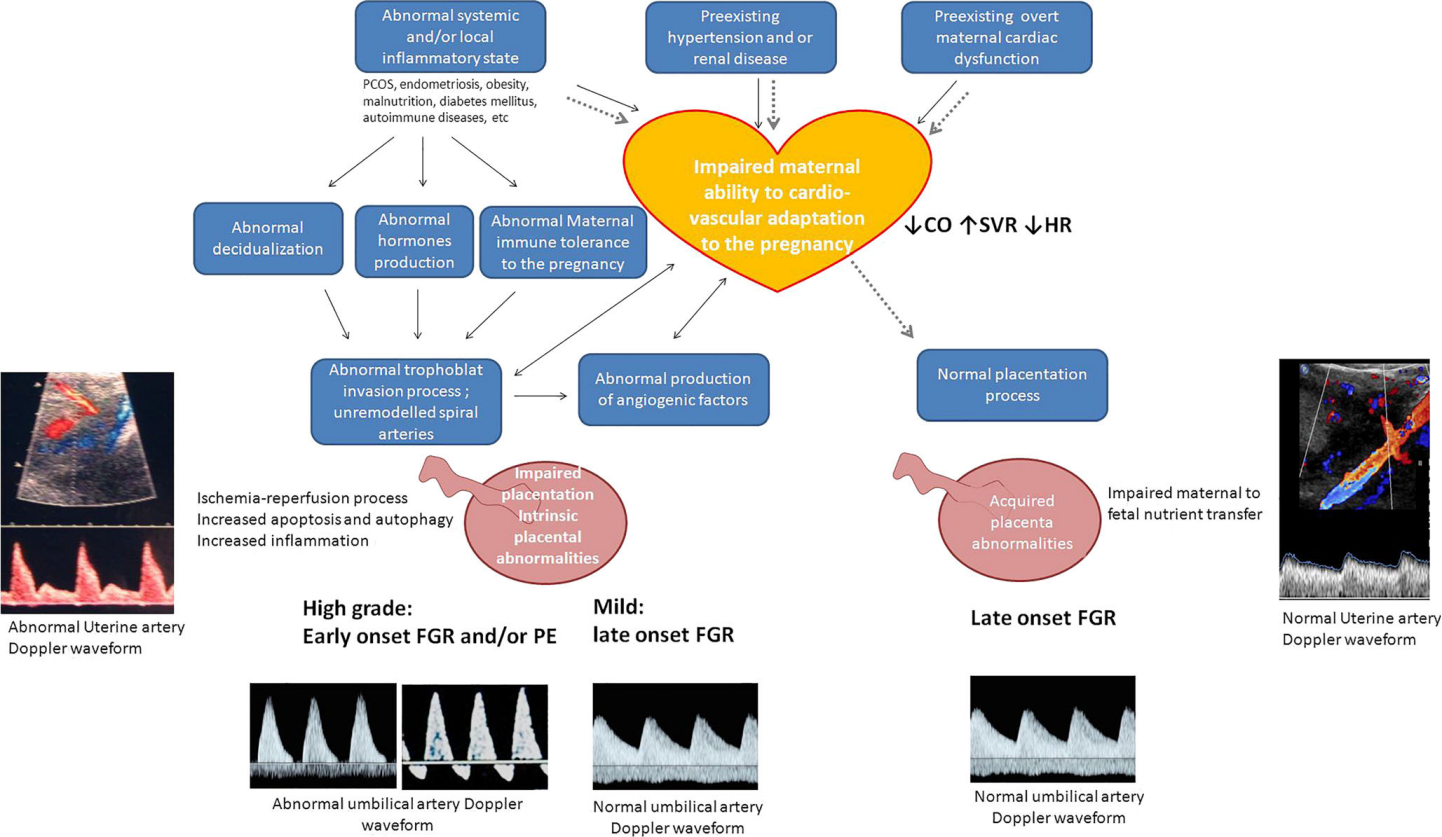
Oriented physiological explanation of the types of FGR according to the maternal cardiac function and placental development. The hypothesis of origin of early-onset FGR (black arrows) and late-onset FGR (gray dotted arrows) is shown. Details are described in the text

The new interesting point of view includes the interaction between placental and maternal cardiovascular system in the pathogenesis of FGR (Fig. 4).

It has been hypothesized that early-onset PE and FGR are due to an association between an intrinsic placental dysfunction with a pre-existent maladaptation of the maternal cardiovascular system. Instead, late-onset fetal growth disorders are more likely to be associated with an acquired placental dysfunction as a result of a static hemodynamic system not being able to meet the excessive demands of an advanced or overgrown pregnancy [11, 72].

A better understanding of maternal hemodynamics in pregnancies complicated by FGR could bring various benefits in clinical practice. In fact, it could have potential therapeutic implications, improve screening tools, help distinguish SGA from FGR, and prevent future CVD in these women. However, further studies are needed to verify if the changes in hemodynamic parameters among FGR women are already present in pre-gestational period and to examine the possibility of manipulating maternal hemodynamics to improve pregnancy outcomes.
